# EMPOWERING AN AGE-FRIENDLY HEALTH CARE WORKFORCE: AN INTERPROFESSIONAL PROGRAM APPROACH

**DOI:** 10.1093/geroni/igae098.3356

**Published:** 2024-12-31

**Authors:** Leah Tobey-Moore, Melanie Beehler, Kathryn Neill, Karen Dickinson, Laura Spradley, Robin McAtee

**Affiliations:** University of Arkansas for Medical Sciences, Little Rock, Arkansas, United States; University of Arkansas for Medical Sciences, Little Rock, Arkansas, United States; University of Arkansas for Medical Sciences, Little Rock, Arkansas, United States; University of Arkansas for Medical Sciences, Little Rock, Arkansas, United States; University of Arkansas for Medical Sciences, Little Rock, Arkansas, United States; University of Arkansas for Medical Sciences, Little Rock, Arkansas, United States

## Abstract

Growing interest in caring for the geriatric population is a challenge in healthcare education. In Arkansas (AR), 18% of the population is over 65. The Arkansas Geriatric Education Collaborative (AGEC) is a HRSA-funded Geriatric Workforce Enhancement grant recipient with a main goal to encourage health professions students toward careers in geriatrics. AGEC’s Geriatric Student Scholar Program exists to expand exposure and foster interest regarding older adults (OA) through experiential learning and interprofessional training. Each year, 4-5 students are chosen to further their knowledge and interest in the field of geriatrics. This year’s team of 5 students (pharmacy, physician assistant, medical doctor, audiology, and physical therapy) completed two geriatric-focused didactic events, two experiential community events, and an interprofessional education (IPE) team project. Their 2024 IPE project focused on Mobility from the 4Ms Framework of Age-Friendly Healthcare and was delivered via an active learning “escape room.” Reflection essays were submitted following each attended activity. AGEC activities supported students’ understanding and application of interprofessional, collaborative skills needed to care for OAs and allowed students to take on the role of educator by creating, designing, and evaluating a learning activity called “Falls and Fall Prevention in the OA.” Programmatic surveys at the conclusion of the 7-month experience demonstrated 1) positive overall experience, 2) enhanced interprofessional collaboration skills, 3) strong teamwork dynamics and 4) deepened interest in geriatrics.

